# Increasing Antimicrobial Resistance of *Vibrio cholerae* OI Biotype EI Tor Strains Isolated in a Tertiary-care Centre in India

**DOI:** 10.3329/jhpn.v30i1.11270

**Published:** 2012-03

**Authors:** Jharna Mandal, K.P. Dinoop, Subhash Chandra Parija

**Affiliations:** Department of Microbiology, Jawaharlal Nehru Institute of Post Graduate Medical Education and Research, Puducherry, India

**Keywords:** Antibiotic resistance, Cholera, Ceftriaxone, Ciprofloxacin, Drug resistance, Microbial, Tetracycline, *Vibrio cholerae*, India

## Abstract

The antimicrobial susceptibility patterns are on constant change with the recent emergence of multidrug-resistant strains of most bacteria. Results of recent studies in India showed that most isolates of *Vibrio cholerae* O1 were resistant to the commonly-used antibiotics. The study was conducted to determine the antibiotic susceptibility patterns of *V. cholerae* O1 isolated during 2008-2010 at the hospital of the Jawaharlal Nehru Institute of Post Graduate Medical Education and Research, Puducherry, India. In total, 154 strains of *V. cholerae* O1 from 2,658 stool specimens were reported during January 2008–December 2010—34 in 2008, 2 in 2009, and 118 in 2010. The isolates of *V. cholerae* O1 were subjected to antimicrobial susceptibili-ty testing using the Kirby-Bauer method. The antibiotic disks tested were tetracycline (30 μg), furazolidone (100 μg), ampicillin (10 μg), ceftriaxone (30 μg), and ciprofloxacin (5 μg). *Escherichia coli* ATCC 25922 was used as the control organism. The minimum inhibitory concentrations (MICs) of ceftriaxone, ciprofloxacin, and tetracycline were determined using the agar dilution method for all the strains. The E-test method was used for the strains which had either intermediate resistance or were resistant to the antibiotics by the agar dilution method. The results of the agar dilution corroborated the results of the E-test. The MIC of ceftriaxone in 151 strains was <2 μg/mL while it was 16 μg/mL in three strains; the latter three strains were resistant to ceftriaxone by the disc-diffusion test. The MIC of ciprofloxacin in 150 strains was <0.5 μg/mL while the MIC of tetracycline was <1 μg/mL. In the remaining four strains, the MIC of tetracycline was >32 μg/mL, and the MIC of ciprofloxacin was >8 μg/mL. These four strains were resistant to both tetracycline and ciprofloxacin by the disc-diffusion test and were exclusive of the three ceftriaxone-resistant strains. The majority of the isolates were obtained from children aged 0-5 year(s)—70.3% (83 of 118) and 41.2% (14 of 34) were reported in 2010 and 2008 respectively. Since treating severe cases of cholera with antibiotics is important, the continuing spread of resistance in *V. cholerae* to the most important agents of therapy is a matter of concern. Also, chemoprophylaxis with antimicrobial agents is likely to become even more difficult.

## INTRODUCTION

*Vibrio cholerae* O1, the causative organism of epidemic cholera, continues to be a major health problem in most parts of developing nations in the world. The outbreaks of cholera follow a seasonal pattern in regions of endemicity, reflecting the shifts in climatic conditions that lead to preferential selection of hosts by the causal organisms (1-3). The replacement of fluid and electrolytes plays a major role in treating cases of cholera. Antibiotics as well play a major role in reducing the shedding of the bacillus, thereby preventing the spread of the disease, in treating severe illness by reducing the volume of diarrhoea, and also reducing the duration of illness and hospitalization (4).

The antibiotics commonly used in the treatment of cholera include tetracycline and fluoroquinolones, such as ciprofloxacin, among others. The antimicrobial susceptibility patterns of *V. cholerae* O1 strains from newly-infected patients are on constant change following the recent emergence and spread of multidrug-resistant strains (1-6). Results of recent studies in India showed that most isolates of *V. cholerae* O1 were resistant to the commonly-used antibiotics, such as ampicillin, furazolidone, ciprofloxacin, and tetracycline (4,6). Resistance of *V. cholerae* to ceftriaxone has been reported from Argentina (7) and recently from Delhi, India (8).

There is no report regarding resistance to the third-generation cephalosporins from our region, this being the first. Also, no consolidated data are available on the antimicrobial susceptibility patterns of the organism concerned. The present study was conducted to determine the antibiotic susceptibility patterns of *V. cholerae* O1 isolated during 2008-2010 at the hospital of the Jawaharlal Nehru Institute of Post Graduate Medical Education and Research (JIPMER), Puducherry, India.

## MATERIALS AND METHODS

The study was conducted at the Department of Microbiology, JIPMER, Puducherry, India. The study samples included strains of *V. cholerae* O1 obtained from 2,658 consecutive stool samples of patients with cholera-like disease during January 2008–December 2010.

The stool samples were inoculated onto MacConkey agar and thiosulphate-citrate-bile salts-sucrose agar (HIMEDIA, Mumbai). Enrichment was done with alkaline peptone water, from which subculture was performed after six hours of incubation at 37 °C onto MacConkey agar and thiosulphate-citrate-bile salts-sucrose agar. Suspected colonies were identified as *V. cholerae* by the standard biochemical tests (9-11), and the serogroup was confirmed by agglutination with specific antiserum (BD DifcoTM, Becton Dickinson, Sparks, Maryland, USA). Antimicrobial susceptibility testing was done using the Kirby-Bauer method (12-14). The antibiotic disks tested were tetracycline (30 μg), furazolidone (100 μg), ampicillin (10 μg), ceftriaxone (30 μg), and ciprofloxacin (5 μg). *Escherichia coli* ATCC 25922 was used as the control organism. The minimum inhibitory concentrations (MICs) of ciprofloxacin, ceftriaxone, and tetracycline were determined by the agar dilution method (14,15) and the E-test (BioMe´rieux) method. The MICs of ceftriaxone, ciprofloxacin, and tetracycline were determined by the agar dilution method for all the strains. The E-test method was used for the strains which had either intermediate resistance or were resistant to the antibiotics by the agar dilution method.

The double-disk synergy test was performed as des-cribed earlier (16) on the three strains which had intermediate resistance to ceftriaxone.

## RESULTS

Of the 2,658 stool samples obtained from patients, 154 isolates of *V. cholerae* were reported during January 2008–December 2010, of which 34 were reported in 2008 and 2 in 2009. These cases were usually documented in May to August of every year. Subsequently, 118 cases were reported in 2010 as there was an outbreak of cholera in Puducherry.

The majority of the reported isolates were from children aged 0-5 year(s)—70.3% (83 of 118) and 41.2% (14 of 34) were reported in 2010 and 2008 respectively (Table 1). Analysis of the common serotypes isolated during the outbreaks of 2008 and 2010 revealed that Ogawa accounted for 97.1% (33 of 34) and 97.5% (113 of 118) of the cases in 2008 and 2010 respectively (Table 2). Oral rehydration therapy was administered to these patients as the primary treatment, followed by antibiotics, such as doxycycline or ciprofloxacin.

There was a significant difference [p<0.0001, 95% confidence interval (CI) 0.9290-0.9975] in the resistance pattern of the strains isolated in 2010 and 2008 for ampicillin. There was also a significant difference (p<0.0001; 95% CI 0.024-0.1175) in the resistance pattern of the strains isolated in 2010 and 2008 for furazolidone. The resistance patterns for ciprofloxacin and tetracycline did not have any significant difference but had a marginal increase (p=0.5; 95% CI 0.000-0.5217 and p=0.49; 95% CI 0.043-0.348 respectively).

Three strains were resistant to ceftriaxone while four were resistant to tetracycline and ciprofloxacin simultaneously by the disc-diffusion method (Table 3). As only two strains were isolated in 2009, these were not included in the table. Both these strains were sensitive to all the antibiotics tested.

The MIC of ceftriaxone in the 151 strains was <2 μg/mL while it was 16 μg/mL in three strains; the latter three strains were resistant to ceftriaxone by the disc-diffusion test. The MIC of ciprofloxacin in the 150 strains was <0.5 μg/mL while the MIC of tetracycline was <1 μg/mL. In the remaining four strains, the MIC of tetracycline was >32 μg/mL, and the MIC of ciprofloxacin was >8 μg/mL. These four strains were resistant to both tetracycline and ciprofloxacin by the disc-diffusion test and were exclusive of the three ceftriaxone-resistant strains. The double-disk synergy test was negative.

## DISCUSSION

The emergence and the spread of resistance to antibiotics among Gram-negative organisms have been increasing rapidly in recent years. The epidemiological importance of preventing these drug-resistant strains from spreading in the community has become a global problem (6). *V. cholerae* is a potent pathogen which can be transmitted easily in the community by the faeco-oral route, in the existing sanitary systems, particularly in developing countries. In 2008, 88% of the population in India had access to an improved water source but only 31% had access to improved sanitation. In rural areas where 72% of the population of India lives, the shares are 84% for water and only 21% for sanitation. In urban areas, 96% had access to an improved water source and 54% to improved sanitation. Access has improved substantially since 1990 when it was estimated to stand at 72% for water and 18% for sanitation. With all these combined and considering the potential of this organism in causing epidemics, the emergence of resistance to antibiotics in such a scenario can have serious effects.

**Table 1.** Distribution of cholera cases in different years

Age (years)	2010		2009		2008	
	No.	%	No.	%	No.	%
0-5	83	70.3	1	50	14	41.2
6-15	11	9.3	1	50	6	17.6
>15	24	20.4	0	-	14	41.2
Total	118	-	2	-	34	-

**Table 2.** Distribution of serotypes of *Vibrio cholerae* isolated in different years

Serotype	2010		2009		2008	
	No.	%	No.	%	No.	%
Ogawa	115	97.5	2	100	33	97.1
Inaba	3	2.5	0	50	1	2.9

**Table 3.** Comparison of antibiotic resistance patterns of isolates by the disc-diffusion method in different years

Year	Strains isolated	Ampicillin		Ciprofloxacin		Ceftriaxone		Furazolidone		Tetracycline	
		No.	%	No.	%	No.	%	No.	%	No.	%
2010	118	97	82.2	5	4.2	3	2.5	112	95	22	18.6
2008	34	2	5.9	0	-	0	-	7	21	4	12
Total	154	99	64.3	5	3.2	3	2	119	77.3	26	16.9

After the 1980s, plasmid-encoded high-level resistance to tetracycline, ampicillin, and sulphonamides was reported from different parts of the world. Subsequently, nalidixic acid became the drug of choice for the empirical treatment of gastroenteritis but this has soon met the same fate as of its prede-cessors (17-20). The widespread prophylactic use of certain antibiotics, such as tetracycline and co-trimoxazole, and their ease of availability over-the-counter have added to the selective pressure for the emergence of multiple antibiotic-resistant *V. chole-rae* (MARV) (17, 21-23).

Following the emergence of *V. cholerae* O139 in June 1992, there has been a marked variability in the susceptibility patterns of O1 strains, with the reporting of a higher proportion of MARV strains (22,23). Continued surveillance revealed a different resistance pattern wherein the strains were more frequently resistant to tetracycline and ampicillin. The emerging strains possessed extra genetic elements which indicate that significant genomic changes have occurred (22). Recent strains have shown an increasing trend of resistance to fluoroquinolones (23,24). These findings suggest that there has been substantial mobility of genetic elements in *V. cholerae*, which could have contributed to the emerging drug resistance.

In this study, children aged less than five years were more commonly involved (Table 1). The majority of the isolates were obtained from children aged 0-5 year(s)—70.3% (83 of 118) and 41.2% (14 of 34) were reported in 2010 and 2008 respectively. This finding should be taken into consideration as the use of antibiotics in these age-groups is already limi-ted by contra-indications.

The predominance of the Ogawa serotype (n=33; 97.1% and n=115; 97.5% respectively) in 2008 and 2010 (Table 2) suggests that it is currently the predominant circulating serotype in this part of the country. The seasonal association of cases was observed. Most cases occurred in May, and the clustering of cases was observed till August. In 2010, there were two outbreaks—one in May and the other one just after November. This clustering of cases can be referred to the rainfall during these months. The strains belonging to the initial outbreak were similar in their antibiotic susceptibility patterns to the second outbreak, with a pattern of ampicillin>furazolidone>tetracycline>ciprofloxacin; all the strains were sensitive to ceftriaxone. Their similar antibiotic susceptibility pattern probably points to their related clonality. Molecular studies are being planned to understand the relatedness of these strains. Results of analysis of the available antimicrobial susceptibility patterns of *V. cholerae* in 2008 and 2010 (Table 3) revealed that the organism has rapidly gained resistance to antibiotics to which it was previously sensitive. The antibiotic susceptibili-ty pattern observed in this study is indicative of an increasing trend of resistance in the strains isolated. Similar findings have been reported elsewhere (4,18-20,23).

The World Health Organization recommends the use of either doxycycline or ciprofloxacin as the treatment of choice (17). Since the treatment of severe cases of diarrhoea is important, the emergence of resistance of *V. cholerae* to the most important agents of therapy for cholera is a matter of concern. Further molecular studies on these strains are being planned.

The negative results of the double-disk synergy test indicate that strains with intermediate resistance to ceftriaxone are not Class A extended-spectrum ß-lactamase (ESBL) producers but may be Class C ESBL producers or may possess some chromosomal mutation. Due to the lack of a genetic analysis of these strains, the exact nature of the underlying mechanisms mediating the reduced susceptibility of the strains to ceftriaxone is not clear. The causes of resistance to third-generation cephalosporins are (a) ESBL of any of the classes, (b) efflux pumps, and (c) chromosomal mutation. Reduced susceptibility to cephalosporins is chromosomally mediated, and the mechanisms resemble those for chromosomally-mediated penicillin resistance. An earlier study on an unrelated Gram-negative pathogen *Neisseria gonorrhoeae* demonstrated that the presence of mosaic *penA allele, mtrR, penB*, and an unknown mutation was the important determinants for conferring intermediate resistance to expanded-spectrum cephalosporins, such as ceftriaxone and cefixime (25). A detailed genetic study of the drug-resistant strains is warranted in this regard.

### Conclusions

Antimicrobial resistance has attained the importance of a global public-health problem. The increase in the magnitude of bacterial species resistant to multiple antimicrobial agents relies on various factors apart from the environmental stresses which the organism is facing over the years. The availability of over-the-counter drugs and widespread institution of irrational chemoprophylaxis before the patient reaches a tertiary-care hospital in developing countries play an important role in the emergence of the expanding resistance patterns to the organism (23,26). Oral tetracycline and fluoroquinolones, such as ciprofloxacin, have been and are being used extensively and misused for a number of infective conditions other than chole-ra in our region. This indiscriminate use of such antimicrobials will soon result in their becoming ineffective against this organism. The current scenario regarding drug resistance in *V. cholerae* is a matter of concern. Due to the rapid emergence of resistance to the empirically-given antibiotics for the treatment of cholera, therapeutic options are limited. This problem becomes more pronounced when the issue of treating cholera in special groups, such as children, women in the antenatal period, and lactating mothers arises. Thus, the antimicrobial profile of organisms, such as *V. cholerae*, should be under constant surveillance to prevent any dangerous epidemic arising from such drug-resistant strains.
